# Detection of the DNA binding of transcription factors *in situ* at the single-cell resolution in cultured cells by proximity ligation assay

**DOI:** 10.1016/j.xpro.2023.102692

**Published:** 2023-11-02

**Authors:** Babul Moni Ram, Chengkai Dai

**Affiliations:** 1 Mouse Cancer Genetics Program, Center for Cancer Research, National Cancer Institute-Frederick, Frederick, MD 21702, USA

**Keywords:** Cell Biology, Cell Culture, Single Cell, Cancer, Genomics, Microscopy, Molecular Biology, Gene Expression, Molecular/Chemical Probes

## Abstract

Transcription factors (TFs) play a pivotal role in gene expression, and their DNA binding is the prerequisite to instigating gene transcription. Here, we present a protocol that exploits the proximity ligation assay technique to measure the DNA-binding activities of TFs *in situ* at the single-cell resolution. We describe steps for immunostaining with specific antibodies against double-stranded DNA and the TFs of interest, probe incubation, proximity ligation, and signal amplification. We then detail procedures for imaging and image analysis.

For complete details on the use and execution of this protocol, please refer to Dai et al. (2015)^1^ and Xu et al. (2023).^2^

## Highlights

• Adaptation of the proximity ligation assay to visualize protein-DNA interactions
• *In situ* single-cell measurement under physiological conditions
• A versatile protocol applicable to numerous DNA-binding proteins

## Abstract (editor highlights)

Publisher’s note: Undertaking any experimental protocol requires adherence to local institutional guidelines for laboratory safety and ethics.

## Graphical abstract

**Figure:**
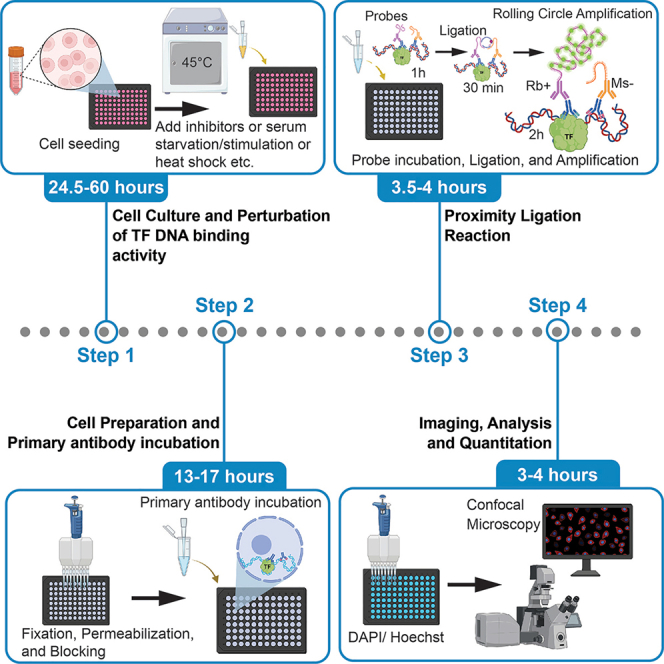

## Before you begin

Transcription is the first step to converting genetic information from DNAs to functional products, such as proteins. One of the central players in transcription are transcription factors (TFs), which dictate gene transcription through direct binding to specific DNA sequences in the genome. To investigate the DNA binding of TFs, a variety of techniques have been developed, including Electrophoretic mobility shift assay (EMSA) and Chromatin Immunoprecipitation (ChIP).^3^^,^^4^ Nonetheless, some limitations are associated with these widely applied techniques, including lengthy procedures, complex non-physiological manipulations, and the requirement for large amounts of samples. Importantly, these techniques can only provide information on the population mean.

The protocol below describes the steps of adapting the Proximity Ligation Assay (PLA) technique to detect the DNA binding of TFs *in situ* at the single-cell resolution. As a recently emerged technique, PLA can detect and visualize endogenous protein-protein interactions and post-translational modifications of proteins.^5^^,^^6^ The success of PLA is primarily determined by the closeness of two targets of interest (within 40 nm).^5^^,^^6^ For typical indirect PLA, two primary antibodies raised in two different species and displaying high specificities towards the targets of interest are required. This protocol can be conveniently applied to any cultured cells and, with some modifications, may also be used on frozen or fixed tissues.

### Optimization of primary antibodies

**Timing: 3–5 days**

1. Pre-determine the optimal concentration of primary antibodies for the immunofluorescence applications to minimize background signals.

***Note:*** In most experiments, a dilution of 1:100 in 1X antibody diluent provided with the Duolink *In Situ* PLA Probe is recommended.

2. Validate the specificities of the primary antibodies being used.
  a. Use genetic or biochemical manipulation of the antigens (e.g., dsDNA and your TF of interest).
  b. Confirm the change by immunofluorescence or immunoblotting to verify the specificity of the antibodies.

***Note:*** Avoid using antibodies with non-specific binding. For example, we combined a mouse monoclonal anti-dsDNA antibody with a rabbit antibody specifically recognizing either c-Myc or heat shock factor 1 (HSF1).^7^^,^^8^ As the validations, we largely abolished the staining of intact cells with the anti-dsDNA antibody by either DNase I digestion of fixed cells or pre-incubation of the antibody with free genomic DNAs.^1^^,^^2^ Similarly, we also markedly diminished the immunoblotting or immunofluorescence signals generated by anti-TF antibodies through genetic depletion of either *c-Myc* or *Hsf1*.^1^^,^^2^

### Culturing cells

**Timing: 24–72 h**

3. For detection by microscopy following PLA, grow the cells on glass slides, chamber slides, or 96-well glass-bottom microplates. Some glass slides require coating them with 0.1 mg/mL Poly-D-lysine solution in sterile water by fully covering the surface for 1 h at 37°C. Troubleshooting problem 1.

***Alternatives:*** Glass slides can also be coated with Collagen I (50 mg/mL in 0.02 N glacial acetic acid) for 1 h at 20°C–22°C.

4. Rinse 3 times with sterile water and allow the slides to dry before seeding the cells.
5. Cell density should be optimized so that cells reach 70%–80% confluence at the time of assay.

### Perturbation of TFs’ DNA-binding activities

**Timing: 0.5–36 h**

6. This technique could, in theory, be applied to determine the DNA binding activity of any TFs. It is important to modulate the DNA binding activity of the TF being studied by genetic, physiological, or pharmacological means.

***Note:*** Genetic knockout or knockdown can be used to deplete the TF. Additionally, pharmacological inhibitors or activators can be used to modulate the function of TF. Moreover, some TF’s activity could be modulated by temperature or hypoxia, etc.

7. Use the TF activity modulated samples as a control to determine and optimize the specificity, effectiveness, and sensitivity of the technique regarding that TF.

### Preparation of reagents

**Timing: 30 min**

8. Fixation Buffer
  a. Prepare a 4% formaldehyde solution in 1X DPBS for use as a fixation buffer.
  b. Store the fixation solution at 4°C for up to 1 month.

**CRITICAL:** Formaldehyde is a hazardous chemical. The fixation solution should be prepared in a chemical fume hood, and gloves and a mask should be used while handling formaldehyde.

9. Permeabilization Buffer
  a. Prepare a 0.3% Triton-X-100 solution in 1X DPBS for use as a permeabilization buffer.
  b. Store the permeabilization buffer at 4°C for up to 1 month.
10. Duolink *In Situ* PLA Fluorescence Wash buffers A and B
  a. Dissolve the contents of one pouch each of Duolink *In Situ* PLA Fluorescence Wash Buffers A and B in distilled water to a final volume of 1 L.

***Note:*** Store the PLA wash buffers at 4°C for up to 1 month. Do not use wash buffers stored longer than a month. Troubleshooting problem 5.

***Alternatives:*** The PLA wash buffers A and B can also be prepared in the lab using the recipe described in the materials and equipment section.

## Key resources table

**Table:** 

REAGENT or RESOURCE	SOURCE	IDENTIFIER
**Antibodies**		

Mouse anti-dsDNA (HYB331-01) antibody (1:100)	Santa Cruz Biotechnology	Cat# sc-58749; RRID: AB_783088
Rabbit anti-c-Myc (D3N8F) antibody (1:100)	Cell Signaling Technology	Cat# 13987S; RRID: AB_2631168
Rabbit anti-HSF1 (D3L8I) antibody (1:100)	Cell Signaling Technology	Cat# 12972S; RRID: AB_2798072
ChromPure rabbit IgG (1:3,000 for c-Myc; 1:10,000 for HSF1)	Jackson ImmunoResearch Laboratories	Cat# 011-000-003; RRID: AB_2337118
ChromPure mouse IgG (1:3,000)	Jackson ImmunoResearch Laboratories	Cat# 015-000-003; RRID: AB_2337188
Duolink *in situ* PLA probe anti-rabbit plus (1:5)	Sigma-Aldrich	Cat# DUO92002; RRID: AB_2810940
Duolink *in situ* PLA probe anti-mouse minus (1:5)	Sigma-Aldrich	Cat# DUO92004; RRID: AB_2713942

**Chemicals, peptides, and recombinant proteins**		

Dimethyl sulfoxide (DMSO)	Sigma-Aldrich	Cat# D2650-100ML
MYCi361	TargetMol Chemicals	Cat# T12132
Tris base	Research Products International	Cat# T60040
Sodium chloride	Research Products International	Cat# S23020
Hydrochloric acid	Acros Organics	Cat# 423790025
Tween 20	Fisher Scientific	Cat# BP337-500
DMEM	Lonza	Cat# 12-604Q
HyClone bovine growth serum	Thermo Scientific	Cat# SH30541.03
Antibiotics (penicillin/streptomycin) 100X	Lonza	Cat# 17-602E
Sodium pyruvate 100X	Gibco	Cat# 11360-070
1X DPBS	Gibco	Cat# 14190
0.05% Trypsin EDTA	Gibco	Cat# 25300-054
Poly-D-lysine hydrobromide	Sigma-Aldrich	Cat# P6407
Formaldehyde, 37%	Fisher Scientific	Cat# F79-1
Triton X-100	Fisher Scientific	Cat# BP151-100
Hoechst 33342	Thermo Fisher Scientific	Cat# 62249
Fluoromount-G	SouthernBiotech	Cat# 0100-01
Duolink *in situ* wash buffers, fluorescence	Sigma-Aldrich	Cat# DUO82049

**Critical commercial assays**		

Duolink *in situ* detection reagents red	Sigma-Aldrich	Cat# DUO92008

**Deposited data**		

Unprocessed microscopy images	This paper	Mendeley Data: https://doi.org/10.17632/x53jgjnsdm.1

**Experimental models: Cell lines**		

Immortalized *Rosa26-CreERT2*; *Hsf1*^*fl/fl*^ MEFs	Su et al.^9^	N/A

**Software and algorithms**		

ImageJ: Fiji	National Institutes of Health	https://imagej.net/software/fiji/
Zeiss Zen (blue edition)	Carl Zeiss Microscopy, LLC	N/A
Prism 9	GraphPad Software	https://www.graphpad.com/scientific-software/prism

**Other**		

ProPlate multi-well chambers	Grace Bio-Labs	Cat# 248860
96-well glass bottom plate with high performance #1.5 cover glass	Cellvis	Cat# P96-1.5H-N
Microscope glass slides (sterile)	Fisher Scientific	Cat# 12-550-15
Microscope coverslips	Fisher Scientific	Cat# 12-541-025
0.22 μm Syringe filter	Pall Corporation	Cat# 4612
Tabletop microcentrifuge	Eppendorf North America	Cat# 5418R
Vortex-Genie 2	Scientific Industries	Cat# SI-0236
Rocking platform shaker	VWR	Cat# 10127-876
CO_2_ incubator	Sanyo	Cat# MCO-18AIC

## Materials and equipment

### Buffer recipes

• 4% Formaldehyde: Add 1.08 mL of 37% formaldehyde to 8.92 mL of 1X DPBS to prepare 10 mL of fixation buffer.

***Note:*** Store the fixation buffer at 4°C for up to one month.

**CRITICAL:** Formaldehyde is a hazardous chemical. The fixation solution should be prepared in a fume hood, and gloves and a mask should be used while handling formaldehyde.

• 0.3% Triton X-100: Add 30 μL of Triton X-100 to 9.97 mL of 1X DPBS to prepare 10 mL of permeabilization buffer.

***Note:*** Store the permeabilization buffer at 4°C for up to one month.

• PLA Wash Buffer A, pH 7.4

**Table:** 

Reagent	Final concentration	Amount
NaCl	150 mM	8.8 *g*
Tris Base	10 mM	1.2 *g*
ddH_2_O	N/A	800 mL
Tween 20	0.05%	0.5 mL
**Total**		**1000 mL**

***Note:*** Adjust the pH to 7.4 using 1 M HCl before adding Tween 20. Filter the solutions using a 0.22 μm filter. Store the buffers at 4°C for up to one month.

• PLA Wash Buffer B, pH 7.5

**Table:** 

Reagent	Final concentration	Amount
NaCl	100 mM	5.84 g
Tris Base	20 mM	24.23 g
ddH_2_O	N/A	800 mL
**Total**		**1000 mL**

***Note:*** Adjust the pH to 7.5 using 1 M HCl. Filter the solutions using a 0.22 μm filter. Store the buffers at 4°C for up to one month.

• Poly-D-lysine solution: dissolve 5 mg of poly-D-lysine in 50 mL of sterile ddH_2_O.

***Note:*** Prepare fresh or store at −20°C for up to one month.

• Complete culture medium:

**Table:** 

Reagent	Final concentration	Amount
DMEM (with 4.5 *g*/L glucose and L-Glutamine)	N/A	880 mL
Bovine growth serum	10%	100 mL
Sodium pyruvate (100X)	1X (1 mM)	10 mL
Penicillin-Streptomycin (100X)	1X (1%)	10 mL
**Total**		**1000 mL**

***Note:*** Prepare the complete culture medium and add the supplements according to the growth medium recommended for the cell line being used. Store the complete media at 4°C until the expiration date.

### Microscopy and imaging

We used a Zeiss LSM780 confocal laser scanning microscope (Zeiss Group, Germany) for imaging in two different fluorescence channels: DAPI for nuclear staining and Texas Red for PLA signals. Microscopy and imaging were controlled using the Zeiss Zen Blue software (Zeiss, Oberkochen, Germany). Images were acquired at 63X magnification at 450 nm and 561 nm wavelengths for DAPI and Texas Red channels, respectively.

### Image analysis

The images, acquired by the LSM780 confocal microscope, were saved as CZI files and processed using the Zeiss Zen Blue software. To quantify PLA signals, Fiji/ImageJ (Version 1.54f) and Java image acquisition plugins were used.

## Step-by-step method details

### Culturing and manipulation of cells

**Timing: 24.5–60 h**

The following steps describe the procedures of culturing mouse embryonic fibroblasts (MEFs) and perturbing the DNA binding activities of c-Myc and HSF1 in MEFs. To study c-Myc, we treat cells with MYCi361, a small molecule disrupting the MYC-MAX dimerization that is essential for the c-Myc DNA binding.^10^ Conversely, we apply serum stimulation to promote the DNA binding of c-Myc.^11^ For the HSF1 study, we subject cells to heat shock, which is a classical stressor to induce the DNA binding of HSF1.^12^

1. Cell culture and modulation of the DNA binding activities of c-Myc and HSF1.
  a. Seed 5×10^3^ *Hsf1* wild type (*Hsf1*^*WT*^) or conditional knockout (*Hsf1*^*CKO*^) MEFs per well in a 96-well glass-bottom microplate in 100 μL of complete DMEM. Allow the cells to grow for 24 h at 37°C in a CO_2_ cell culture incubator.
  b. To prepare serum stimulation, replace complete culture media by serum-free media for 12 h.
    i. Stimulate serum-starved cells by media supplemented with 20% serum for 24 h.
    ii. For the control, serum-starve cells for 12 h without the subsequent serum stimulation.
  c. To block the c-Myc DNA binding, treat cells with 20 μM MYCi361 for 24 h. For the control, treat cells with DMSO.
  d. To induce the HSF1 DNA binding, culture cells at 45°C for 30 min. For the control, culture cells at 37°C.

### Preparation of cells for PLA

**Timing: 1 h**

This step describes the fixation, permeabilization, and blocking procedures necessary for the subsequent incubation with primary antibodies.

2. Cell fixation, permeabilization, and blocking.
  a. Using a vacuum pump or micropipette, aspirate the media from the wells of the 96-well glass-bottom microplate and rinse the cells once with 150 μL 1X DPBS.
  b. Add 100 μL of the fixation solution in each well and incubate for 10–15 min at 20°C–22°C. Avoid more than 15 min fixation with 4% formaldehyde. Troubleshooting problem 5.
  c. Aspirate the fixation solution using a vacuum pump or micropipette and rinse once with 150 μL 1X DPBS.
    **Pause point:** After fixation, the cells can be stored in 1X DPBS for up to one week at 4°C.
  d. Add 100 μL of the permeabilization buffer and incubate for 10 min at 20°C–22°C.
  e. Discard the permeabilization solution using a vacuum pump or micropipette and rinse once with 150 μL 1X DPBS.
  f. Add 50 μL of the blocking solution provided with the Duolink *In Situ* PLA probe kits. Incubate the samples in a 37°C humidified incubator for 30 min.
    ***Alternatives:*** A 5% normal goat serum diluted in 1x DPBS can be used for blocking.

### Proximity ligation assay (PLA)

**Timing: 16–20 h**

This major step includes incubating the samples with the primary antibodies and performing the proximity ligation reaction. After blocking, the cells are incubated with the primary antibodies followed by probe incubation, ligation, and amplification reactions.

***Note:*** The multiple washing steps in this major step must be carried out gently on a slow rocker, as some cell lines detach easily. Troubleshooting problem 2. The wash buffers should be made freshly or stored at 4°C for less than one month. Troubleshooting problem 5.

3. Primary antibody incubation.
  a. Dilute the primary antibodies to optimized concentrations in the antibody dilution buffer provided with the Duolink *In Situ* PLA probe kits. Use the same concentrations of normal rabbit and mouse IgG as the negative controls.
    ***Note:*** We use a 1:100 dilution of each antibody (i.e., 2 μg/mL mouse anti-dsDNA antibody, 4.29 μg/mL rabbit anti-c-Myc, or 1 μg/mL rabbit anti-HSF1 antibody).
  b. Remove the blocking solution from the wells and add 100 μL of the diluted primary antibodies to each well. Incubate for 12–16 h on a slow laboratory rocker at 4°C.
    ***Note:*** Primary antibodies could also be incubated at 20°C–22°C for 1–4 h or at 37°C for 1–2 h. However, the incubation times need to be optimized for individual antibodies. Troubleshooting problem 5.
    **CRITICAL:** The two primary antibodies being used must be raised in two different species. Troubleshooting problem 3.
4. PLA Probe incubation.
  a. Discard the primary antibodies from the wells and gently wash the cells with 150 μL of Wash Buffer A on a slow laboratory rocker for 5 min at 20°C–22°C. Repeat the washing step twice for 2 min each.
  b. Dilute both the 5X Duolink I*n Situ* PLA anti-Rabbit PLUS and 5X Duolink I*n Situ* PLA anti-Mouse MINUS probes together in the 1X antibody diluent provided in the kit to make a 1X working probe dilution.
    **CRITICAL:** The probe mix must include a PLUS PLA probe with another MINUS PLA probe targeting two separate species corresponding to the primary antibodies (e.g., an anti-rabbit PLUS probe should be combined with an anti-mouse MINUS probe). Troubleshooting problem 3.
  c. Discard the Wash Buffer A from the wells using a micropipette and add 40 μL of the diluted PLA probes to each well.
    ***Note:*** It is important to fully cover the entire sample with the diluted probes. If needed, the total volume in each well could be increased to 50–60 μL.
  d. Incubate the plate for 1 h at 37°C in a humidified incubator.
    ***Alternatives:*** A CO_2_ cell culture incubator could be used.
5. Oligonucleotide ligation reaction.
  a. Discard the probes and wash the cells by adding 100 μL of Wash Buffer A on a slow laboratory rocker at 20°C–22°C for 5 min. Repeat the washing step twice for 2 min each.
  b. Dilute the 5X Ligation Buffer provided with the Duolink *In Situ* PLA probe kits to 1X working solution using purified molecular biology grade water. Add 1 μL of the DNA Ligase per 39 μL of the 1X Ligation buffer to prepare the ligation reaction mix.
    ***Note:*** The 5X ligation buffer should be fully thawed and resolved on ice before dilution. It is important to store the ligase in a −20°C freezer and remove it right before adding it to the reaction mix. Troubleshooting problems 3 and 4.
  c. Add 40 μL of the ligation reaction mix to each well and incubate the plate at 37°C for 30 min in a humidified incubator.
    ***Alternatives:*** A CO_2_ cell culture incubator could be used.
6. Rolling circle amplification reaction.
  a. Discard the ligation reaction mix from the wells and wash the cells by adding 100 μL of Wash Buffer A on a slow laboratory rocker for 5 min at 20°C–22°C. Repeat the washing step twice for 2 min each.
  b. Dilute the 5X Amplification Buffer provided with the Duolink *In Situ* Detection Reagents kit to 1X working solution using purified molecular biology grade water. Add 0.5 μL of the DNA polymerase to prepare the amplification reaction mix in 39.5 μL of the 1X Amplification buffer.
    ***Note:*** The amplification buffer should be fully thawed and resolved before dilution. The DNA polymerase should be stored in a −20°C freezer and removed just before adding it to the reaction mix. If possible, aliquot the DNA polymerase and avoid repeated freeze/thaw cycles. Troubleshooting problems 3 and 4.
  c. Add 40 μL of the reaction mix to each well and incubate the plate for 100–120 min at 37°C in a humidified incubator.
    ***Alternatives:*** A CO_2_ cell culture incubator could be used.
    ***Note:*** The amplification reaction is light sensitive, so the samples should be covered and protected from light during the amplification reaction. Some weaker interactions may require longer incubation durations. The incubation timings need to be optimized based on the TFs being studied. Troubleshooting problems 3 and 4.

### Mounting and confocal microscopy

**Timing: 2–3 h**

This major step involves nuclear counterstaining, mounting and image acquisition using confocal microscope.

7. Nuclear staining and mounting.
  a. Discard the amplification reaction mix from the wells and wash the cells by adding 100 μL of 1X Wash Buffer B on a slow laboratory rocker for 5 min at 20°C–22°C. Wash two more times using 0.01X Wash Buffer B for 2 min each.
  b. Prepare 2 μM Hoechst 33342 solution for nuclear staining in molecular biology grade water.
  c. Add 100 μL of the nuclear staining solution in each well and incubate on a slow laboratory rocker for 5 min at 20°C–22°C.
  d. Discard the nuclear staining solution and wash the cells twice with molecular biology grade water for 2 min each on a slow laboratory rocker at 20°C–22°C.
  e. Air dry the cells for 2 min at 20°C–22°C and add 60 μL of the Fluoromount-G mounting medium to each well. In the case of multi-well chambers, mark the wells on the reverse side of the glass slides using a marker before removing the chamber for air drying and mounting. Add a coverslip to the slide and avoid air bubbles.

***Alternatives:*** Instead of nuclear staining using Hoechst 33342, a mounting medium with DAPI can be used. In that case, directly proceed to step e after step a. After adding the mounting medium with DAPI, add the coverslip and seal the edges of the coverslip using clear nail polish.

**Pause point:** The plate or slide may be stored at 4°C protecting from light for up to two weeks before imaging.

8. Confocal microscopy and imaging.
  a. Set up the acquisition parameters depending on the fluorophores and the confocal microscope used. Troubleshooting problem 3.
  b. Use a 20X lens to first locate the cells on the slide and signal distribution. Use a 63X lens for the acquisition of PLA signals.
  c. Use the IgG control samples to adjust the photomultiplier tube (PMT) settings for acquiring the images. Acquire images using the DAPI channel (405 nm) and Texas Red (561 nm) for nuclear staining and PLA red signal, respectively.

***Note:*** We use the Duolink *In Situ* Detection Reagents Red for our experiments. Use the appropriate channel depending on the PLA detection kit being used for the experiment (e.g., use the FITC or Alexa Fluor 488 channel for the Duolink *In Situ* Detection Reagents Green).

**CRITICAL:** The PMT parameters should be set using the IgG controls and kept uniform across all the samples imaged for data comparison. Use immersion oil for the 63X lens and the coverslip should be facing the lens. Follow proper instructions for operating the confocal microscope.

## Expected outcomes

**Figure 1:**
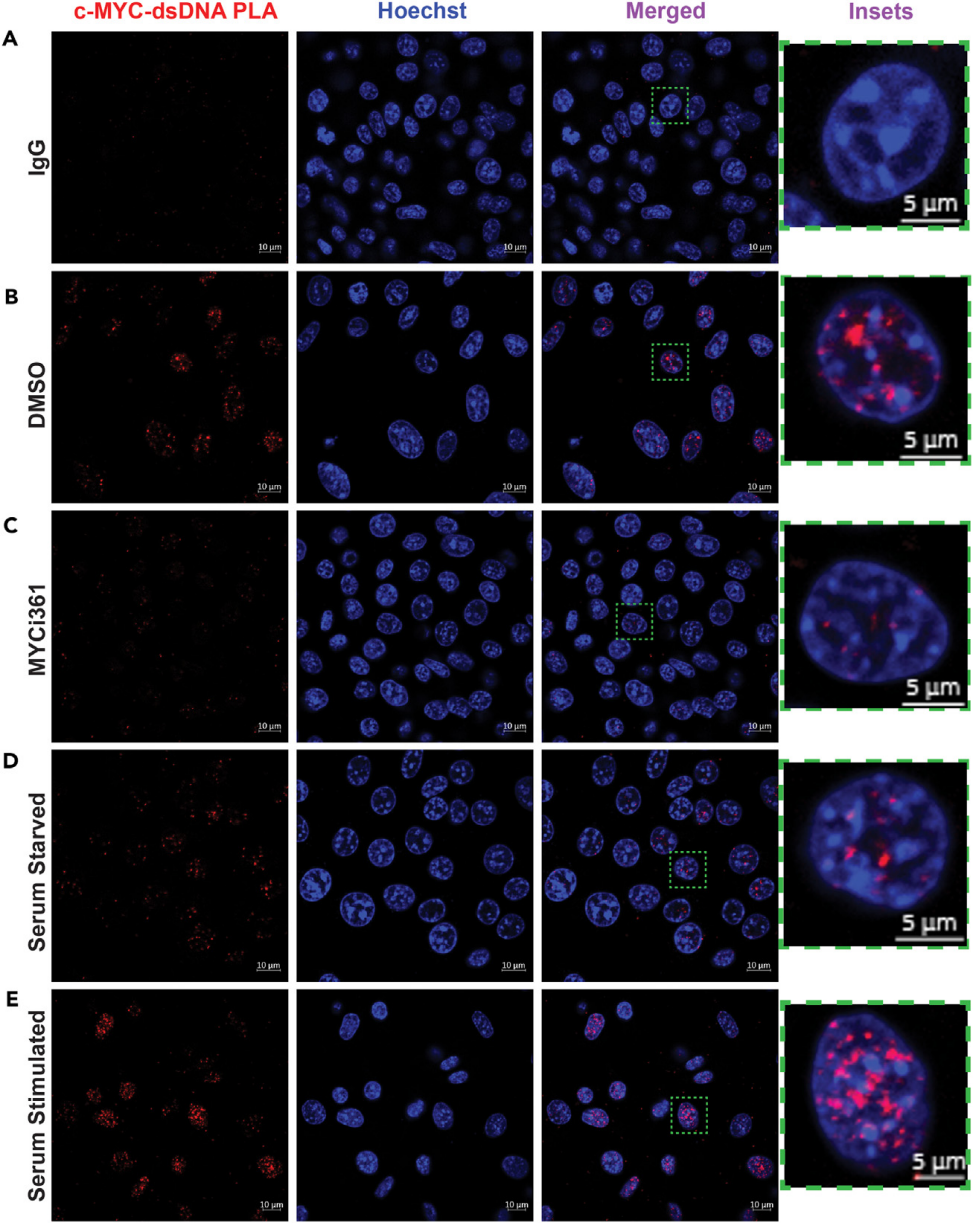
c-Myc binds to genomic DNAs in *Hsf1*^*WT*^ MEFs (A) Negative controls for PLA using normal mouse IgG and rabbit anti-c-Myc antibodies. (B) Visualization of endogenous c-Myc binding to genomic DNAs by PLA (red) in MEFs treated with DMSO. (C) Decreased PLA signals in MEFs treated with 20 μM MYCi361 for 24 h. (D) PLA signals in MEFs serum starved for 12 h. (E) PLA signals in MEFs stimulated with 20% serum for 24 h following a 12-h serum starvation. Scale bars: 10 μm. Inset scale bars: 5 μm.

**Figure 2:**
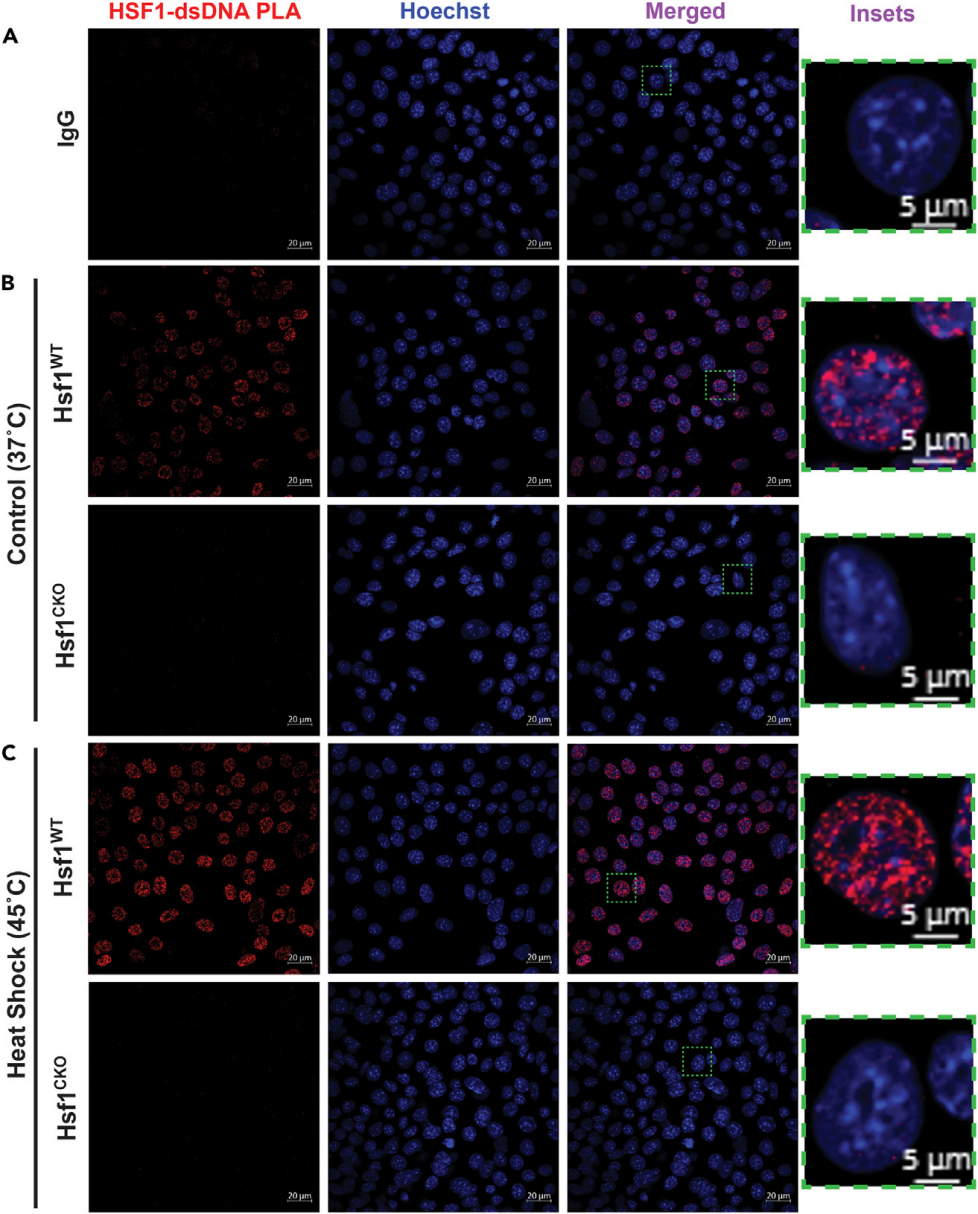
HSF1 binds to genomic DNAs in *Hsf1*^*WT*^ MEFs (A) Negative controls for PLA using normal rabbit and mouse IgG. (B) Visualization of endogenous HSF1 binding to genomic DNAs by PLA (red) in *Hsf1*^*WT*^ and *Hsf1*^*CKO*^ MEFs respectively under normal (37°C) conditions. (C) Visualization of endogenous HSF1 binding to genomic DNAs by PLA (red) in *Hsf1*^*WT*^ and *Hsf1*^*CKO*^ MEFs respectively under heat shock (45°C) conditions. Scale bars: 20 μm. Inset scale bars: 5 μm.

PLA has been employed as a means to study *in situ* protein-protein interactions. Here, we present a simple and effective PLA-based protocol for visualizing and quantifying endogenous TF-DNA interactions at the single-cell resolution. Compared to the technical IgG controls, DNA binding of c-Myc can be visualized as red fluorescent foci co-localized with the Hoechst 33342 nuclear staining (Figures 1A and 1B). As further biological validations, treatment with MYCi361, which disrupts the MYC-MAX dimerization, markedly diminished the PLA signals. Similarly, serum starvation decreased while serum stimulation enhanced the PLA signals (Figures 1C–1E), as expected. Similarly, heat shock heightened the HSF1-dsDNA PLA signals in *Hsf1*^*WT*^ MEFs (Figures 2B and 2C), congruent with HSF1 activation by heat shock.^6^ Importantly, these PLA signals were absent in *Hsf1*^*CKO*^ cells (Figures 2B and 2C). In conclusion, this protocol described here can accurately detect the DNA binding of endogenous TFs at the single-cell resolution.

To quantify these PLA signals, we applied the Fiji image processing software to count the number of fluorescent foci. The data are represented as the number of PLA foci per nucleus for statistical comparisons.

## Quantification and statistical analysis

The images acquired using the Zeiss Zen Blue software were saved as CZI files. The display parameters (i.e., brightness and contrast of acquired images) can be adjusted to the same levels as the IgG control images if required, and scale bars are applied. The images were exported as TIFF files.

1. Open the CZI files in the Fiji software for quantitation as split channels (Figure 3A).

**Figure 3:**
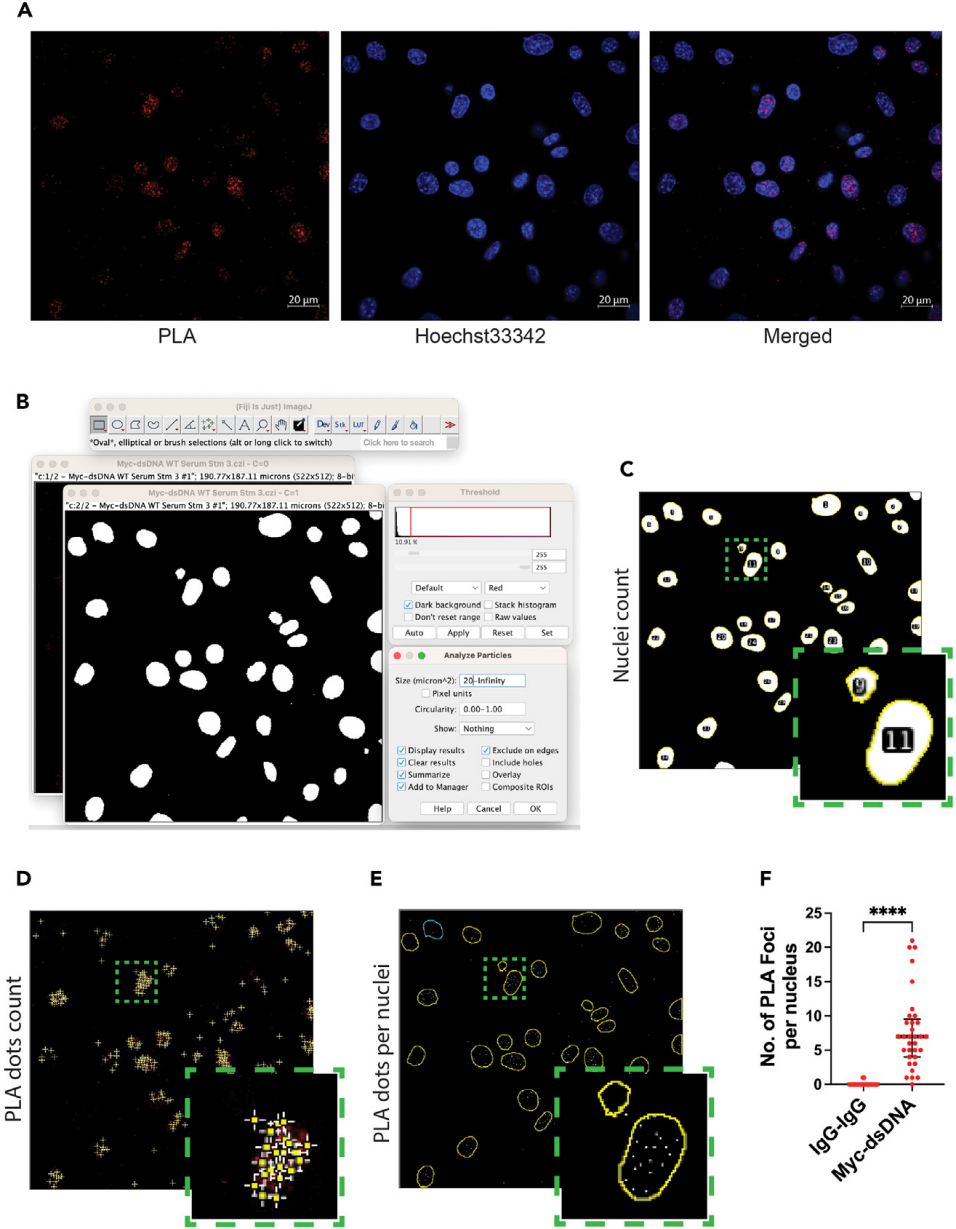
Experimental workflow for quantitation of PLA foci using the Fiji/ImageJ software (A) Split channel images of c-Myc DNA binding PLA showing the separate PLA and Hoechst 33342 channels. Scale bars: 20 μm. (B) Adjustment of the threshold value to select individual nuclei for quantitation. (C) Quantitation of the number of nuclei in the image. (D) Quantitation of the fluorescent foci representing the PLA signals. (E) Binary image showing the PLA signal in each nucleus. (F) Quantitation of the number of PLA foci per nucleus. ∗∗∗∗p < 0.0001 (median ± IQR, n = 99 or 33 nuclei, Mann Whitney U test).
2. Select the DAPI channel and adjust the threshold using *Image/Adjust/Threshold* to an optimal value so that each nucleus is highlighted as a single region. We set a threshold value of 25 (Figure 3B).
3. Set size discrimination using *Analyze/Analyze particles* to exclude the smaller objects that are not nuclei. We set a minimum value of 20 μm^2^ and a maximum of infinity (Figure 3B). After pressing OK, the results are displayed as an image with nuclear count (Figure 3C) and in a separate table.
4. Select the PLA channel image to count the number of foci. Adjust the prominence using *Process/Find maxima* and select *Single point output type* (Figure 3D). Click OK to generate a binary image with a single pixel for each local maximum.
5. Open the ROI manager using *Analyze/Tools/ROI Manager* and check *Show All.* This gives a merged image of PLA pixels and the nuclear area (Figure 3E).
6. Measure from the ROI manager menu to obtain a table of results. In the results, the *RawIntDen* represents the sum of all pixels in that region. Each pixel represents a maximum with a value of 255. Therefore, dividing each *RawIntDen* value by 255 gives the total number of foci in that region defined by the nuclear stain earlier. The final obtained values can be plotted as the number of PLA foci per nucleus (Figure 3F).

**CRITICAL:** All the parameters set in the Fiji software during analysis and quantitation should be kept uniform across all the images used for data comparison.

## Limitations

Compared to other common techniques, such as EMSA and ChIP, this *in situ* PLA-based technique is convenient and can be performed under physiological conditions, entailing simple experimental procedures without cell disruption. Nevertheless, there are some limitations. While this PLA-based technique is straightforward to detect and visualize global DNA binding of TFs, it could not pinpoint which specific genomic loci TFs bind to, since the anti-dsDNA antibody does not recognize specific DNA sequences. However, this limitation may be overcome by incorporating the capability of the CRISPR-Cas9 system to target specific DNA sequences. The selection of primary antibodies remains another limitation of this protocol, as antibody combinations raised in two different species are required when performing indirect PLA, which relies on the usage of probes conjugated with secondary antibodies. The availability of target-specific and validated antibodies raised at different species is limiting. However, this may be overcome by directly conjugating the oligonucleotide probes to primary antibodies. Beyond cultured cells, this protocol should also be applicable to tissues, although tissue autofluorescence is likely problematic. Nonetheless, this may be overcome by using the bright-field detection reagent, which is based on the horseradish peroxidase (HRP)/3,3′-diaminobenzidine (DAB) system.

## Troubleshooting

### Problem 1

The cells are not properly adhered to the glass slides (related to before you begin: step 3).

### Potential solution

Use 0.01% Poly-D-lysine or Poly-L-lysine solution or 50 mg/mL Collagen I to coat the slides before seeding cells.

### Problem 2

A low number of cells during imaging (related to step-by-step method details: step 3–6).

### Potential solution

Add the wash buffers gently during washing steps as cells get detached during washing. Use a slow rocking platform during the washing steps as some cell lines detach easily while handling.

### Problem 3

No PLA signals (related to step-by-step method details: step 3–8).

### Potential solution

• Make sure to add all the correct components at the primary antibodies’ incubation, probe incubation, ligation, and amplification steps.
• Use proper fluorescence acquisition parameters and PMT settings during confocal microscopy based on the PLA detection kit used.

### Problem 4

Low PLA signals (related to step-by-step method details: step 5–6).

### Potential solution

• Make sure that the ligase and polymerase are properly stored at −20°C as they are sensitive to temperature fluctuations.
• Increase the amplification time as some weak interactions require prolonged amplification time.
• Avoid light exposure during and after the amplification step.
• Follow manufacturer’s instructions for storage of components.

### Problem 5

High background signals or diffused signals (related to step-by-step method details: step 2–6).

### Potential solution

• Do not fix samples in formaldehyde for more than 15 min, as it will increase autofluorescence.
• Carefully follow the blocking and washing steps.
• Use optimized antibody concentrations and incubation duration and temperatures.
• Prepare fresh PLA wash buffers A and B. Do not use expired or old PLA wash buffers.
• Follow the manufacturer’s instructions for the storage and handling of components being used.

## Resource availability

### Lead contact

Further information and requests for resources and reagents should be directed to and will be fulfilled by the lead contact, Chengkai Dai (chengkai.dai@nih.gov).

### Materials availability

This study did not generate new unique reagents.

## Data Availability

This study did not generate original code. The original microscopy images reported in this study have been deposited at Mendeley and are publicly available as of the date of publication. The DOI is listed in the key resources table.
